# Brain dynamic functional connectivity in patients with disorders of consciousness

**DOI:** 10.1186/1471-2202-15-S1-P105

**Published:** 2014-07-21

**Authors:** Verónica Mäki-Marttunen

**Affiliations:** 1 Department of Neuroimaging, FLENI, Buenos Aires 1428, Argentina; 2 CONICET, Buenos Aires, Argentina

## 

Ever since the proposal that brain activity is organized into networks of coherent activity in the framework of functional connectivity (FC, [1]), a great amount of research has been conducted to characterize these networks, their inter-relations, and their differential participation in sensory-motor and cognitive processes, as well as their alteration in brain states such as pathological diseases, sleep, etc. The main techniques to study FC are functional magnetic resonance imaging (fMRI) and positron emition tomography (PET), but it has also been described using other techniques, such as MEG and EEG [2]. The classic approach to obtain FC networks is to calculate the correlation between the low-pass filtered time series of brain nodes (i.e., voxels, anatomical areas, etc.) along the scanning period. In this way the studies on FC commonly assume a rather static correlation pattern. Although such studies unraveled important features of brain large-scale functioning, FC is in fact highly variable in time and across subjects [3]. Recent work digs deeper into this variability and brings up important caveats to many previous assumptions. For instance, new findings suggest that networks may reorganize in time, weakening their intra-network connectivity strengh and enforcing the inter-network connectivities, and that there may exist discrete states of multistability over time [4].

In the present work the dynamic functional connectivity was analyzed in a group of patients with disorders of consciousness (DOC) as well as in healthy subjects, during a resting state (RS) fMRI paradigm. The correlation matrices (**R**) between predefined brain areas were obtained in sequential time windows. The temporal variability of different metrics of the **R** matrices was examined. Furthermore, main components (i.e., hidden states of frequent brain configurations) were identified from DOC or Control groups' **R** temporal series by employing singular value decomposition (SVD). SVD is a dimensionality reduction technique that allows for obtaining a latent correlation structure model, and has been previously applied by Leonardi et al. [5] to RS fMRI data. In the present work we investigated whether the main SVD components differ between DOC and Control groups and in which temporal manner. Preliminary results show that the main components present dissimilarities between the groups in specific brain areas. Furthermore, the temporal variation of the largest eigenvalue (that is interpreted as the collective motion of all areas) of the **R** matrices is different in DOC compared to Control group.

The results may contribute to the understanding of the process of consciousness in so far as brain dynamic functionality and have potential impact in the understanding of consciousness disorder.
